# Community-level Monitoring of Antimicrobial Resistance following Mass Administration of Azithromycin to Reduce Child Mortality in Côte d’Ivoire: Results from Two Rounds of Surveillance

**DOI:** 10.12688/gatesopenres.16385.1

**Published:** 2026-06-02

**Authors:** Lisa Dulli, Valérie Gbonon, Fatoumata Touré, Daouda Touré, Bertin Guédé Kipré, Felicity Nelson, Kate Murray, Emily Evens, Mireille Dosso, Pat Sadate-Ngatchou

**Affiliations:** 1 FHI 360, Durham, North Carolina, USA; 2 Institut Pasteur de Cote d'Ivoire, Abidjan, Cote d'Ivoire; 3 FHI 360, Abidjan, Cote d'Ivoire; 4 Universite Peleforo Gon Coulibaly, Korhogo, Cote d'Ivoire; 5 PnP Ahead LLC, Seattle, Washington, USA

**Keywords:** Antimicrobial resistance (AMR), Mass drug administration (MDA), Azithromycin, Child mortality reduction, Streptococcus pneumoniae

## Abstract

**Background:**

Mass Drug Administration (MDA) of azithromycin for the elimination of trachoma and, more recently, for the reduction of child mortality, are public health strategies shown, with mixed results, to contribute to the development of azithromycin resistance in treated communities. The objective of this study was to monitor bacterial resistance to azithromycin among children targeted by bi-annual MDA-azithromycin campaigns to improve child survival in eight high-mortality regions of Côte d’Ivoire.

**Methods:**

Two rounds of cross-sectional surveys were carried out in targeted health districts using a 2-stage cluster sampling to select representative samples of children aged 1–11 months at the time of the most recent MDA. Parents/primary caregivers were interviewed about the child’s overall health and exposure to MDA, azithromycin, and other antibiotics. Nasopharyngeal swabs were collected to estimate the carriage of *Streptococcus pneumoniae* and the prevalence of macrolide-resistant *S. pneumoniae* using erythromycin as a proxy for azithromycin. Phenotypic and genotypic determinants of macrolide resistance were tested in a subsample of resistant bacteria using duplex PCR.

**Results:**

A total of 606 and 692 eligible children in 2022 and 2023, respectively, were included in the analyses. Prevalence of *S. pneumoniae* carriage was 28.8% (95%CI:22.4, 35.3) in 2022 and 30.8% (95%CI:25.0, 36.7) in 2023 (p=0.6506). There was no statistically significant difference in the prevalence of erythromycin-resistant *S. pneumoniae* between the two surveys (32.8% (95%CI:25.3, 40.2) versus 26.2% (95%CI:19.8, 32.6); p=0.0537). The majority of macrolide resistance phenotypes in both surveys were predominantly constitutive macrolide-lincosamide-streptogramin B (MLSb), carried by erm(B)-type genes.

**Conclusions:**

Community-level bacterial resistance to azithromycin in our samples of *S. pneumoniae* did not increase significantly between the two surveys. As countries weigh the benefits and costs to implement MDA azithromycin, more is needed to interpret the relevancy and clinical impact of observed antimicrobial resistance.

## Introduction

Antimicrobial resistance (AMR) is a global public health threat. Researchers have estimated that AMR was associated with nearly 5 million deaths in 2019, including over 1 million deaths associated with resistant bacterial infections. ^1^ If nothing is done to abate this, by 2050, it is estimated that AMR will lead to 10 million deaths and cost approximately $100 trillion (USD). ^2^

Mass drug administration (MDA) of azithromycin (MDA-AZM) for the prevention and treatment of ocular *chlamydia trachomatis* (trachoma), and more recently, for the reduction of child mortality in high-mortality settings, are public health strategies that are feared to contribute to the development of azithromycin resistance in treated communities, as well as possible resistance to macrolide antibiotics more broadly and potentially to the transfer of resistance to other organisms. ^3^ Several studies have examined the effects of MDA-AZM on the development of bacterial resistance to the drug over the past decade, and results from these studies have been mixed. Some studies have found evidence of transient resistance in some bacteria, while others have found no evidence of bacterial resistance following MDAs. A few studies have demonstrated that post-MDA resistance may be determined by pre-MDA levels of resistant bacteria in MDA targeted communities. ^4^^–^^19^

Two relatively recent (2015 and 2019) systematic literature reviews have summarized the evidence provided by this body of research. Ho et al. (2015) evaluated eight studies that examined the effects of MDA-AZM on the carriage and subsequent development of resistance to the drug in *Streptococcus pneumoniae (S. pneumoniae).* ^18^ According to the authors, five of the eight studies found low levels of baseline resistance to azithromycin in communities targeted for MDA; in three of these five studies, resistance increased shortly after drug distribution but dropped considerably over time, returning to near baseline by 12 months after the MDAs. Two studies revealed high baseline resistance levels that were sustained at six months after the MDAs; however, in both studies, MDAs were carried out at greater frequencies than in other research settings (biannually or quarterly). The authors concluded that communities with low levels of baseline resistance to azithromycin experienced only transient increases in resistance; however, communities with higher levels of resistance, which were also correlated with more frequent MDA events, were more likely to experience sustained higher levels of resistance.

O’Brien et al. updated the Ho review in 2019 and expanded it to include studies that examined resistance to other pathogens in addition to *S. pneumoniae.* The authors identified 18 published studies and one unpublished dataset that examined the effect of MDA-AZM on the development of resistance in communities for the following organisms: *S. Pneumoniae (n = 12), Staphylococcus Aureus (n = 3), Chlamydia trachomatis (n = 3), Escherichia coli (n = 3)*, and *Plasmodium falciparum (n = 1).* MDA frequency varied across studies from annual distribution to multiple times per year; follow-up periods also varied considerably from 2 weeks to 4 years. Six studies included pre-MDA baseline assessments and five others included control communities for comparison. Of the studies that examined resistance in *S. pneumoniae*, findings were consistent with the previous review: resistance increased transiently after MDAs and returned to near baseline after some time. Among the three studies that examined *E. coli*, all three found increased levels of resistance immediately after, and six months post MDA. For the two studies that examined resistance in *S. aureus*, neither study had a control group or baseline assessment; however, both studies revealed that resistance in this organism increased over time following MDAs. Lastly, no studies have found evidence of the development of resistance in either *C. trachomatis* or *P. falciparum* following MDA of azithromycin.

More recently, analyses of data from the MORDOR ( *Macrolides Oraux pour Réduire les Décès avec un Oeil sur la Résistance*) trials have been reported. ^20^ One study noted that after two years of bi-annual MDA-AZM for children, the prevalence of resistant strains of *S. pneumoniae* to both erythromycin and clindamycin was significantly greater in azithromycin-treated communities than in untreated control communities, as was macrolide resistance among gut bacteria from rectal swabs; however, there were no differences between the two groups in resistance to other classes of antibiotics. ^11^ A second study on the effect of MDA-AZM on gut biomes in preschool children in Niger demonstrated that prolonged MDA-AZM was associated with reductions in certain gut bacteria, including pathogens, as well as increases in both macrolide and non-macrolide resistance in treated communities as compared to non-treated control communities. ^12^

### AMR surveillance in the context of MDA-AZM

Based on the available evidence, the World Health Organization (WHO) released guidance in 2020 on the implementation of bi-annual MDA-AZM to reduce child mortality. ^3^ Specifically, it was recommended that the intervention be implemented only in high mortality settings, where the infant mortality rate (IMR) exceeded 60 deaths per 1,000 live births or the under-5 child mortality rate (U5MR) exceeded 80 deaths per 1,000 live births. ^3^ Additionally, the WHO guidance specifically noted that implementing MDA-AZM should be accompanied by continuous AMR monitoring. Specifically, the guidelines call for sentinel surveillance at the community level for “resistance of nasopharyngeal flora (Streptococcus pneumoniae and Streptococcus pyogenes), gut flora (Salmonella spp., Shigella spp., and Enterobacteriaceae), and common bacteria causing invasive infections.” ^3^

As part of the Resiliency through Azithromycin for Children (REACH) project, an implementation research study designed to examine the scale-up of in high-child mortality regions in Côte d’Ivoire was carried out from 2021 to 2024. During this time, the Government of Côte d’Ivoire, through the National Program for the Fight against Neglected Tropical Diseases (NTDP), delivered four rounds of MDA-AZM to 19 health districts (August 2021, April 2022, December 2022, July 2023). The 19 health districts were identified as having high child mortality, consistent with WHO guidance, and were planned to receive MDA-AZM targeting the general population for trachoma prevention. After the first round of MDA-AZM for the entire population that took place in August 2021, subsequent rounds of MDA-AZM targeted only children aged 1 to 11 months and specifically as part of the REACH program. Two additional rounds of MDA-AZM were delivered by the National Nutrition Program (NNP) in conjunction with vitamin A mass distribution events in November 2023 and June 2024. The health districts covered by the NNP overlapped somewhat with those targeted earlier by the NTDP, but not completely.

The study team worked closely with the government of Côte d’Ivoire and the Institut Pasteur of Côte d’Ivoire (IPCI) to plan and implement activities to monitor antibiotic resistance trends in the 19 health districts targeted by the NTDP for MDA-AZM. These plans included supporting the addition of azithromycin to the panel of drugs tested for resistant infections identified through the IPCI’s sentinel surveillance program, as well as the implementation of annual, cross-sectional community-based antimicrobial resistance (AMR) surveillance surveys in a probability sample of communities targeted by the REACH project. This paper reports the results of the first two rounds of community-based AMR surveillance studies conducted in Côte d’Ivoire.

### Ethics statement

This study was reviewed and approved by the Côte d’Ivoire National Ethics Committee for Life and Health Sciences (Abidjan, Côte d’Ivoire) *(study number 047-22/MSHPCMU/CNESVS-kp)* and the FHI 360 Protection of Human Subjects Committee (Durham, NC, USA) ( *study number 1868409).* All methods were carried out in accordance with relevant guidelines and regulations.

## Methods

### Overview

The REACH project conducted two rounds of community-based AMR surveillance among representative samples of households with children eligible to receive azithromycin during the most recent MDA-AZM. This periodic surveillance was designed to describe patterns and changes in AMR over time, rather than to estimate a causal effect of MDA-AZM on AMR. A planned round of surveillance prior to the beginning of the intervention did not take place because it corresponded to the onset of the COVID-19 pandemic.

Prior to participation, written informed consent to participate in the study was obtained from all parents/guardians who took part in the survey portion of the study and written parental permission was obtained to obtain the nasopharyngeal sample from each child participant. In each sampled household, a parent/guardian of the eligible child was interviewed to collect data on basic sociodemographics, the child’s participation in the MDA, and other antibiotic exposure in the previous 30 days. The questionnaire was followed by the collection of a nasopharyngeal swab from the eligible child to test for *Streptococcus pneumoniae (S. pneumoniae)* bacterium. Specimens that were positive for *S. pneumoniae* were further tested for phenotypic resistance to a panel of antibiotics including azithromycin. A subset of specimens that demonstrated resistance to erythromycin (a proxy for azithromycin resistance) was tested for genomic resistance.

The MDAs were implemented twice yearly from 2021 to 2023 in 19 health districts in Côte d’Ivoire, which had been identified as meeting the WHO-recommended threshold for child or infant mortality. An average of approximately 159,000 children between 1 and 11 months of age (inclusive) in 19 health districts received a single dose of azithromycin for each MDA. AMR surveillance surveys were conducted 3 months after the MDAs were conducted in April 2022 and July 2023.

A two-stage, cluster random sample of households was selected for each round of surveillance, in which census enumeration areas (EAs) served as the primary sampling unit and households the secondary sampling unit sampled probability proportional to size, with the total EA population serving as the measure of size and stratified by zone (north, northeast, and west). To calculate the sample size, we need to first estimate the prevalence of bacterial carriage for *S. pneumoniae* among the target population, and then estimate the proportion of those who carry resistant bacteria. In the absence of baseline estimates of the prevalence of bacterial carriage or AMR in the Côte d’Ivoire health regions targeted by REACH, we assumed that approximately 50% of children would be colonized with the targeted bacteria, and 50% of those bacteria would be resistant to azithromycin, resulting in an estimated prevalence of resistant bacteria of 25% among all children sampled, which is consistent with the prevalence reported by similar studies in the region. ^6^^,^^15^ Assuming a 95% confidence interval with a width of 0.1, a moderate ICC of 0.05, and a possible non-response rate of 10%, the total sample size was 748 child participants, or 17 children from each of 44 clusters, resulting in a design effect of 1.70. Within the sampled EAs, a study team accompanied by local community health workers mapped the EA and identified households with eligible children to create a sampling frame for households from which a random sample of 17 households was selected in each EA. If a household contained more than one eligible child, one was randomly selected for participation using an algorithm programmed into the computer tablet.

A research assistant interviewed the parents using a brief structured questionnaire, and a trained laboratory technician collected the nasopharyngeal swab. Data collected through the questionnaire included sociodemographic information for the parent (sex, age, education), knowledge of the azithromycin MDA for infants and whether the child received the medication at the during the last MDA. The child’s exposure to the MDA and whether or not they received the medication were derived from these parent/caregiver reports. Additionally, parents were asked, for those children who received the medication, if they had experienced any adverse drug reactions, and whether a drug reaction required evaluation or treatment by a health care professional. During the second round of surveillance, questions on infant vaccine history were included. The survey data were collected electronically on computer tablets and uploaded to a server.

### Sample size

The primary aim of these surveys was to estimate the prevalence of bacterial resistance to azithromycin in the targeted communities to allow decision makers to monitor trends in AMR over time. In the absence of existing data on the prevalence of bacterial carriage or AMR in the districts targeted by REACH, we assumed a conservative baseline prevalence of resistant bacteria among the study population to be 50%. Assuming a 95% confidence interval with a width of 0.1, adjusting for cluster effects using a moderate ICC of 0.05 and a possible 10% non-response, the sample size per survey round was 748 parent/child pairs from 44 clusters, resulting in a design effect of 1.70.

### Survey data analysis

All analyses accounted for the two-stage stratified cluster design with EAs as the primary sampling unit (PSU) and stratification by zone (north, northeast, west). Sampling weights reflected the inverse probability of selection at each stage (EA selection PPS and household selection within EA) and were applied in SAS survey procedures. Descriptive estimates and 95% confidence intervals were computed using design-based variance estimation. Comparisons between rounds used survey logistic regression with survey round as the primary predictor.

### Specimen processing and analyses

Nasopharyngeal specimens were collected from each child using a sterile nasopharyngeal collection swab, which was then immediately placed into a sterile tube containing a trans-isolate (TI) medium, labeled, and then placed in a cryogenic transport box. Transport boxes were transferred in no more than 8 hours to a regional laboratory, where the specimens were stored at -20°C until they could be transported to the Institute Pasteur laboratory in Abidjan, where they were stored at -80°C. The process of transporting the biological samples collected during the survey was compliant with the NF EI ISO 15189 standard. ^22^

For bacterial culture, each nasopharyngeal sample was inoculated onto an agar medium containing 5% sheep blood and 5 mg/L gentamycin, and then incubated at 37°C in a 5% CO2 enriched atmosphere using a candle jar for 18-24 hours. *S. pneumoniae* was identified by alpha-hemolysis and the results of three phenotypic/biochemical tests (optochin sensitivity, inulin fermentation, and bile solubility). Each strain of *S. pneumoniae* was subsequently confirmed using an automated identification system (VITEK2 ^®^ /Biomérieux) or by Mass Spectrometry MALDI TOF-MS (BioMérieux, France). Antibiotic susceptibility tests were carried out on all strains by disk diffusion method in an agar medium according to the European Committee on Antimicrobial Susceptibility and the Antibiogram Committee of the French Society of Microbiology guidelines 2021. ^23^

Consistent with guidance from the European Committee on Antimicrobial Susceptibility Testing (EUCAST) and the *Comité de l’Antibiogramme de la Société Française de Microbiologie* (CA-SFM) and others, erythromycin susceptibility was used to determine the susceptibility to azithromycin. ^33^^–^^35^ Clindamycin, a lincosamide and closely related macrolides routinely evaluated as part of AMR surveillance in Cote d’Ivoire, was also tested. Inhibition diameters were measured using ADAGIO ^®^ (BioRad, Marnes, France). The measurement of inhibition diameters was used to categorize the strains into susceptible (S), intermediate (I), and resistant (R) according to the recommendations of EUCAST/CASFM, 2021. ^23^ Minimum inhibitory concentration (MIC) of azithromycin against *S.* *pneumoniae* isolates was determined using the azithromycin E-test (BioMérieux, France). *S. pneumoniae* ATCC 49619 was used as the quality control strain and included in each set of tests to ensure accurate results.

Molecular testing of azithromycin resistance genes was performed on all resistant specimens by duplex PCR for the erm and mef genes according to the method described in the procedure manual of the National Guide for Molecular Detection of Antimicrobial Resistance. ^24^ Positive and negative controls were included in each series. *S. pneumoniae* strains with azithromycin-resistant genes (PCR-positive) were stored at -80°C for subsequent sequencing and in liquid nitrogen (-180°C).

## Results

A total of 606 eligible children from 41 clusters in 2022 and 692 eligible children from 44 clusters in 2023 were included in the study analyses. ^25^ Parent characteristics did not differ significantly across time points ( Table 1). Coverage from MDA-AZM was lower than anticipated (targeted 90% coverage) at both time points but did not differ significantly between the two surveys.

**Table 1.** Respondent characteristics by survey year. Estimates are weighed and adjusted for sampling effects.

Respondent characteristics	2022 AMR Survey % [95%CI] (unweighted n = 606)	2023 AMR Survey % [95%CI] (unweighted n = 692)	p-value
Sex (% Female)	91.6 [85.8, 97.3]	95.8 [93.4, 98.1] ^1^	0.12
Age (mean)	28.2 [26.9, 29.5]	27.5 [26.6, 28.3]	0.35
Education			
None	63.2 [57.0, 69.4]	69.4 [63.3, 75.6]	0.19
Primary school or less	21.6 [16.0, 27.1]	18.0 [14.2, 21.8]	
Any secondary school or higher	15.2 [10.2, 20.2]	12.6 [8.3, 16.8]	
Relationship to child - Parent	95.9 [93.6, 98.1]	96.8 [95.4, 98.3]	0.44
Child age group during previous MDA			
1-5 months	51.2 [46.2, 56.2]	52.5 [48.8, 56.2]	0.68
6-11 months	48.8 [43.8, 53.8]	47.5 [43.8, 51.2]	
Received azithromycin during MDA	65.2 [54.3, 76.0] ^2^	63.1 [53.2, 72.9] ^3^	0.77

^1^ 4 missing.

^2^ 8 missing.

^3^ 49 missing.

The prevalence of *S. pneumoniae* carriage was 28.8% (95% CI:22.4, 35.3) in 2022 and 30.8% (95% CI:25.0, 36.7) in 2023 and did not differ significantly across time points (p = 0.6506) ( Table 2). Resistance to erythromycin was tested as a proxy for azithromycin at both time points, although azithromycin itself was added to the resistance testing panel in 2023. Approximately 32.8% (95% CI:25.3, 40.2) of specimens in 2022 and 26.2% (95% CI:19.8, 32.6) in 2023 were resistant to erythromycin, although the difference between the two timepoints was not statistically significant (p = 0.0537).

**Table 2.** Bacterial culture results and antibacterial resistance testing by survey year for macrolides. Estimates are weighted and adjusted for sampling design.

Characteristics	2022 AMR Survey % [95%CI]	2023 AMR Survey % [95%CI]	p-value
Culture positive for strep pneumonia	28.8 [22.4, 35.3]	30.8 [25.0, 36.7]	0.65
**Among culture positive**	**(n = 179)**	**(n = 193)**	
Erythromycin			
Sensitive	63.0 [54.2, 71.7]	73.2 [66.1, 80.3]	0.0537
Resistant	32.8 [25.3, 40.2]	26.2 [19.8, 32.6]	
Indeterminate	4.3 [0.5, 8.1]	0.6 [0, 1.9]	
Clindamycin			
Sensitive	76.3 [66.6, 85.9]	88.2 [83.3, 93.1]	0.02
Resistant	23.7 [14.1, 33.4]	11.8 [6.9, 16.7]	
Indeterminate	--	- -	
Azithromycin ^1^			
Sensitive	-	74.9 [69.1, 80.6]	
Resistant	-	25.1 [19.4, 30.9]	
Indeterminate	-	-	

^1^ Resistance testing not performed for samples in 2022.

The resistance of *S. pneumoniae* isolates to clindamycin was slightly lower, at 23.7% [14.1, 33.4] in 2022 and 11.8% [6.9, 16.7] in 2023 ( Table 2).

Phenotypic test results for the first survey showed that among the S. pneumoniae isolates examined, 42 (23%) had a constitutive MLSb resistance phenotype and five isolates (2.7%) had an inductive MLSb resistance phenotype. During survey 2, the constitutive MLSb resistance phenotype was present in 24 isolates (12.4%), and 1 isolate (0.5%) had an inductive MLSb resistance phenotype ( Table 3).

**Table 3.** Phenotypic and genotypic resistance testing results.

Characteristics	2022 AMR Survey % (n)	2023 AMR Survey % (n)
**Phenotypic resistance**	n = 183	n = 193
Macrolides		
MLSb ^1^ constitutive	23.0 (42)	12.4 (24)
MLSb ^1^ inductive	2.7 (5)	0.5 (1)
**Genotypic resistance**	n = 35	n = 41
erm(B)+	37.1 (13)	17.1 (7)
mef	0 (0)	0 (0)

^1^ MLSB = Macrolides, Lincosamides, and Streptogramin B.

In 2022, 35 S. pneumoniae isolates with resistance phenotypes were tested using PCR. The ermB resistance gene was detected in 13 isolates (37.1%). In 2023, out of the 41 S. pneumoniae isolates on which PCR was performed, seven (17.1%) isolates contained ermB genes. No mef gene was detected.

## Discussion

The REACH project in Côte d’Ivoire carried out two rounds of community-based surveillance studies over the course of the three-year project in an effort to contribute to monitoring the potential adverse effects of the MDA-AZM intervention in targeted communities. The findings from this study are consistent with the results of other studies that examined AMR among children in MDA-azithromycin trials. Other studies have reported rates of *S pneumoniae carriage* as low as 10-20% and as high as 89-90% in different settings and resistance levels of approximately 10-50% of isolates. ^5^^,^^8^^,^^11^^,^^12^^,^^15^^,^^17^^,^^19^

The aim of this surveillance was not to examine a direct association between MDA exposure and AMR, but rather to document macrolide resistance rates in communities targeted by the MDA-AZM intervention so that changes over time could be monitored as part of routine surveillance for this intervention. Such community-based surveillance can provide important insights into the prevalence of drug-resistant bacteria in these communities. Further sequential routine assessments will permit the analysis of trends in prevalence over time, which will be useful to decision makers as they weigh the potential benefits and risks of the intervention strategy.

Two main mechanisms of macrolide resistance have been described in *S. pneumoniae*: methylation of the 23S ribosomal subunit, which gives rise to the MLSb phenotype (high-level resistance to macrolides, lincosamides, and synergistin B); and an active efflux system, which gives rise to the M phenotype. This is due to the expression of the erm(B) and mef(A) genes, which may be chromosomal or plasmid-mediated.

The expression of erm can be inducible or constitutive. The constitutive phenotype is a permanent expression that makes bacteria resistant to MLSBs and ketolides from the outset. An inducible phenotype requires the presence of an inducing antibiotic to be expressed. The inducers were macrolides with 14, 15, and 16 carbon atoms, lincosamides, and streptogramin B.

The majority of macrolide resistance phenotypes in both surveys were constitutive MLSb carried by erm(B)-type genes. Our results, in line with previous studies that have shown that erm(B) is the most common genotype worldwide and the most common mechanism of resistance to erythromycin, indicate that there may be a higher risk of resistance dissemination if these genes are also plasmid-mediated. ^26^^–^^28^

Although azithromycin is not widely used outside MDAs in Côte d’Ivoire, ^29^ it is an important drug for a few common, severe infections, such as community-acquired bacterial pneumonia, some sexually transmitted infections, and enteric fever. ^30^ Azithromycin is an important treatment option for shigellosis. ^31^ Resistance to erythromycin and azithromycin may also lead to the development of resistance to other antibiotics. ^19^ Decision makers need to reflect on the relevance of AMR surveillance trends and consider a threshold for deciding whether and when to discontinue MDAs if increases in AMR are observed over time.

Despite only having data from two time points, it is initially encouraging that AMR in our *S. pneumoniae* did not increase significantly between the two survey time points; in fact, the absolute values decreased slightly, indicating no preliminary evidence of an upward trend. Exposure to MDA prior to each survey was lower than anticipated.

Community-level surveillance of carriage for specific bacteria and the resistance of these bacteria to various antibiotics in an asymptomatic population can serve as an early warning system in the context of routine mass drug administration programs that distribute antibiotics to community members. It is important that surveillance and routine results review continue to inform actions. Guidance will be needed from stakeholders and AMR experts to define thresholds that would prompt further investigations such as confirmatory sampling, expanded molecular characterization/typing where feasible, and assessment of plausible drivers. Programmatic actions can then be determined in consultation with national guidelines and stakeholders, consistent with the descriptive surveillance purpose of this study.

Given the transient nature of resistance identified in some studies following MDA azithromycin, the long-term clinical relevance of community AMR will also be important. Therefore, establishing or strengthening routine facility-based AMR surveillance to examine trends in infections caused by resistant organisms is also necessary. In Côte d’Ivoire, efforts have been made to add azithromycin to drug sensitivity testing through their regional laboratory network; however, the current passive facility-based AMR surveillance system relies on patients paying for testing when needed. ^32^ As a result, the number of specimens received for AMR testing are very low across the country. For example, in 2022, 3,849 specimens were received by the National Reference Laboratory for AMR testing, 96% (3,712) of which originated from one facility, the University Hospital of Cocody, where IPCI is headquartered in Abidjan. ^31^ Developing strategies and identifying resources to leverage the existing regional network could provide important insights into trends in clinical infections associated with resistant organisms. This information could further help guide decision-makers in monitoring AMR in MDA azithromycin-targeted areas.

### Limitations

Three rounds of surveillance in intervention communities were originally planned for the three-year project; however, the initial round of surveillance was delayed significantly due to the COVID-19 pandemic, leaving only enough time to complete two rounds. The timing of surveillance relative to MDA activities was a function of access to necessary equipment and supplies for the first round of surveillance in 2022 (due to ongoing supply chain disruptions from COVID-19).

Another limitation is that 63 observations from the first round of surveillance were excluded from the analysis because the children did not meet the age eligibility for inclusion. This error was due to the need to calculate the age at time of MDA, which occurred prior to the surveillance study. Additional measures were taken to prevent this during the second round of surveillance. Additionally, during the first round of surveillance, data were collected from 3 EAs (35 observations total) where the MDA-AZM was planned but not ultimately conducted due to programmatic changes. These data were also excluded from the analysis. A sensitivity analysis comparing respondent characteristics, pneumococcal carriage, and resistance to erythromycin showed no significant differences on any variable when these 98 were excluded from the sample.

## Conclusion

MDA-azithromycin is a promising intervention that may help improve child survival. However, we are still learning about the potential long-term consequences of the ongoing mass distribution of this important antibiotic. As countries weigh the benefits and costs of implementing MDA-AZM, the capacity and resources necessary to adequately monitor AMR must be included in these decisions. The availability of necessary laboratory resources and expertise is paramount.

More is needed to interpret the relevance of AMR changes observed in these settings – at what point do changes in antibiotic resistance warrant considering discontinuing the intervention? Therefore, understanding the potential clinical and environmental outcomes associated with mass antibiotic distribution is critical.

## Data Availability

Dataverse: Community-level Monitoring of Antimicrobial Resistance following Mass Administration of Azithromycin to Reduce Child Mortality in Côte d’Ivoire: Results from Two Rounds of Surveillance. https://doi.org/10.7910/DVN/9BAA5H. The project contains the following underlying data:
• For Round 1: REACH_AMR Survey Lab Data_2022 • For Round 2: REACH_AMR Survey Lab Data_2023 The study data and corresponding documentation are available on Harvard Dataverse under the terms of the CC0 1.0 license.
